# Mindfulness is not associated with dissonant attitudes but enhances the ability to cope with them

**DOI:** 10.1186/s40359-020-0377-x

**Published:** 2020-04-10

**Authors:** Carolin Muschalik, Rik Crutzen, Iman Elfeddali, Hein de Vries

**Affiliations:** 1 Department of Health Promotion, Care and Public Health Research Institute (Caphri), Maastricht University, Maastricht, 6200 MD The Netherlands; 2 GGz Breburg, Academic Department of Specialized Mental Health Care, Tilburg, 5000 AT The Netherlands; 3 Tilburg University, Tranzo - Scientific Center for Care and Welfare, Tilburg, 5000 LE The Netherlands

**Keywords:** Implicit attitudes, Explicit attitudes, Implicit-explicit dissonance, Mindfulness, Acceptance, Behavior, Intention

## Abstract

**Background:**

Explicit and implicit attitudes have been studied extensively, but there is less attention to reducing dissonance between them. This is relevant because this dissonance (IED) results in distress and has inconsistent effects on behavior, e.g. less physical activity but more smoking. Mindfulness decreases dissonance between self-related explicit and implicit constructs. This study investigates if, and which, specific mindfulness subskills are associated with decreased dissonance between explicit and implicit attitudes, and whether mindfulness subskills moderate the relationship between IED and intention/behavior.

**Method:**

At baseline and one and three months thereafter, participants’ (*N* = 1476) explicit attitudes, implicit attitudes, red meat consumption (RMC), intention to reduce RMC as well as levels of trait mindfulness were assessed.

**Results:**

Mindfulness subskills were not associated with decreased IED. IED was associated with lower RMC and a higher intention to reduce RMC. The mindfulness subskill acceptance buffered the effect of IED on intention, seemingly offering a skill to deal with dissonant attitudes, which was unidentified until now.

**Conclusion:**

The mindfulness subskill accepting without judgment functions as a way to deal with dissonance. Future research should use this novel finding and investigate whether mindfulness can be used as a buffer in contexts where dissonance results in maladaptive behaviors.

## Background

Dual-process models suggest that individuals have two sources for their evaluative tendencies [1–5]. The first source roots in intentional reasoning and is based on beliefs, which the individual considers as true. These beliefs are presented in explicit constructs towards an object or behavior (e.g. an explicit attitude). The second source draws on intuitive feelings and automatic evaluations towards a target and shapes a person’s implicit tendencies (e.g. an implicit attitude or an approach or avoidance tendency). These rather unconscious evaluations occur regardless of whether the individual considers them as true or not [6]. According to dual-process models, explicit constructs are part of the reflective system. Evaluations within this system are traditionally assessed through self-reports. Automatic evaluations, on the contrary, are part of the impulsive system. These evaluations are inferred by reaction time tasks, such as the Implicit Association Task (IAT; [7]). It is assumed that both types of evaluations can occur simultaneously [2, 8, 9] and that they can be in line with each other (i.e. the explicit and implicit evaluations regarding a subject are both positive or both negative), but also dissonant (i.e. one evaluation is positive whereas the other is negative).

Dissonance between these two types of evaluations has mainly been explored for self-related topics such as self-esteem or affective experiences [10–12]. This dissonance has been shown to result in psychological distress and negative health outcomes, such as unhealthy forms of perfectionism, higher levels of narcissism, being more vulnerable to criticism, being more prone for anger suppression [11, 13–15] as well as in diminished physical and psychological health [14]. Hence, congruence between implicit and explicit evaluations might decrease distress and improve health outcomes.

One factor that has been shown to enhance congruence between dissonant implicit and explicit evaluations is mindfulness [10, 12]. Mindfulness can be understood as “the awareness that emerges through paying attention on purpose, in the present moment, and nonjudgmentally to the unfolding of experience moment by moment” [16]. Mindfulness can be divided into five different facets or subskills: observing, describing, acting with awareness, accept without judgment, and non-reactivity to inner experiences [17, 18]. Observing is the ability to observe, notice, and attend to internal and external phenomena (e.g. bodily sensations, or smells). Describing refers to the ability to describe, label, or note observed phenomena by using words in a non-judgmental way. Acting with awareness concerns the ability to be attentive and fully engaged in one’s present activity, and accepting without judgment is the tendency to accept one’s thoughts and feelings without evaluating them. Non-reactivity to inner experiences is the ability to allow one’s thoughts and feelings to come and go, without getting carried away by them or acting on them. In sum, mindfulness involves the pure perception of one’s internal and external experiences in an objective way. Since this mindful processing enhances the clarity of one’s thoughts, feelings, behaviors, and sensations [19], it may allow intuitive, implicitly represented information about objects (i.e., implicit attitudes) to become incorporated into a person’s conscious experience (i.e., explicit attitude). In addition, mindfulness facilitates the ability to observe one’s (inner) experiences without judgment or attempts to change those. Hence, mindful individuals may also be more acceptive of their implicit attitudes once they become aware of them. We do not assume that implicit attitudes are more ‘correct’ than explicit attitudes, but that mindfulness might help to get in touch with a person’s implicit attitude. This might result in expressing one’s implicit attitude also in an explicit way. Thereby, discrepancy between a person’s implicit and explicit attitude might be decreased.

Also Brown and Ryan [12] concluded that mindfulness can facilitate the uncovering of rather inaccessible (unconscious or automatic) realities. Participants in their study that scored higher on dispositional (trait) mindfulness had a greater congruence between their implicitly and explicitly measured affective experience (i.e. the current experience of positive and negative emotions). Koole et al. [10] revealed that meditation – a practice that aims to promote mindfulness – enhanced the congruence between implicitly and explicitly measured self-esteem. Presumably, mindfulness increased the sensitivity and attention towards inner (rather unconscious) emotions, which are normally less accessible. This insight, in turn, was used to reflect on one’s explicit report and resulted in a more accurate evaluation of self-related constructs, which then resulted in more congruent attitudes. Until now, a few studies have investigated in how far mindfulness enables individuals to decrease the level of dissonance regarding self-related constructs, which are normally highly emotionally laden. Self-esteem, for example, is defined as a “favourable or unfavourable attitude toward the self” [20]. Hence, the self is in the focus of the evaluation. It is unclear, whether mindfulness can also help to decrease the level of dissonance between implicit and explicit attitudes – constructs that are, although also to some degree, less self-relevant and less experiential, but more actionable. An attitude is defined as “a psychological tendency that is expressed by evaluating a particular entity with some degree of favor or disfavor” [21]. Hence, not the self is the focus of the evaluation but another entity. To our knowledge, the question whether mindfulness can decrease dissonance between implicit and explicit attitudes regarding entities other than the self has been unaddressed and builds, therefore, the focus of this study.

As far as we know, only Hyde and colleagues [22] performed a similar study. More precisely, they investigated whether the congruence between implicit and explicit attitudes regarding physical activity is moderated by the private self-consciousness and private body consciousness. No effect was found. Private self-consciousness is defined as the tendency to focus on internal thoughts and feelings [23], and private body consciousness as the ability to be sensitive to internal bodily states [24]. These definitions correspond with the mindfulness subskill observing, which can be defined as the ability to observe, notice or attend to stimuli including internal phenomena (cognitions, bodily sensations) and external phenomena (sounds, smells) [25]. However, it is reasonable to assume that in order to translate one’s implicit attitude into one’s explicit attitude, it is not only necessary to be able to have insight into one’s inner processes (i.e. observing or private body consciousness) but also to accept one’s inner processes as they are (i.e. accepting without judgement). For example, when they are inconsistent with one’s personal belief system or perceived social norms. Additionally, for the translation of implicit attitudes into explicit statements, it might be essential that an individual does not react automatically in case dissonance between his or her implicit and explicit attitudes is detected. For example, by trying to alter his or her inner experiences (i.e. non-reactivity to inner experiences). The exclusive focus on private self-consciousness and private body consciousness (i.e. observing) might be a reason for the null findings in the study of Hyde et al. [22]. Moreover, neither of the aforementioned studies [10, 12] investigated which specific mindfulness subskill(s) lead to a greater congruence between implicitly and explicitly measured constructs, but simply concluded that the ability to have insight into one’s inner processes (i.e. observing) was responsible for that. Consequently, the present study investigated not only whether trait mindfulness is related to the congruence between implicit attitude and explicit attitudes, but also which subskill of trait mindfulness is responsible for this occurrence. As the relations between mindfulness and IED were based on theoretical reasoning and have not been explored before, we investigated our research questions in an exploratory way.

Attitudes are a key construct in social psychology and are regarded as important determinants across various (health) behaviors. Both implicit and explicit attitudes can predict the same (health) behavior [26, 27] and individuals can hold dissonant explicit and implicit attitudes towards the same behavior or object [28–31]. Similarly to dissonant self-relevant constructs, dissonant attitudes are experienced as unpleasant [29] and lead to difficulties in functioning, which individuals are highly motivated to diminish. More importantly, this dissonance has an impact on (health) behaviors [29, 32–34]. Participants with more dissonant implicit and explicit attitudes towards a person were more motivated to engage in additional information processing regarding that person [29], which was presumably an attempt to decrease dissonance. Also, more dissonant implicit and explicit attitudes towards physical activity are associated with lower levels of physical activity [32]. Individuals are assumingly uncertain about whether to move towards the behavior or not. On the other hand, smokers with dissonant implicit and explicit attitudes towards smoking are more likely to resolve this conflict by smoking a cigarette [34] and more dissonant implicit and explicit attitudes result in more disinhibited chocolate consumption [33]. The authors explained that the dissonance intensified the focus on the object (chocolate), which in turn resulted in higher consumption. Moreover Goldstein et al. [33] found the personality trait impulsivity to moderate the effect between the dissonance of attitudes and behavior, in the sense that dissonance was predictive for individuals with high levels of impulsivity but not for individuals with low levels of impulsivity.

The aforementioned studies demonstrate that the effects of implicit-explicit discrepancy (IED) on behavior are inconsistent (i.e. more information processing, less physical activity, more smoking, more disinhibited chocolate consumption). Therefore, the question arises whether the effect of IED on behavior could be moderated by a third variable. Goldstein et al. [33] demonstrated that IED predicted disinhibited eating especially in individuals high in levels of impulsivity. According to Murphy and MacKillop [35] “impulsivity and mindfulness are natural reciprocals” (page 528). Although both constructs entail a focus on the present moment, the present moment is approached differently. That is, impulsivity entails an overemphasis on the present moment, without an adequate reflection on the future or on consequences of one’s action [36]. Mindfulness also entails a focus on the present moment, however, by noticing and experiencing it fully. Hence, it occurs without judgment and reactivity. Therefore, impulsivity reflects a greater likelihood to act on an impulse, and mindfulness reflects a decreased likelihood to do so, which stems from the acknowledgment of impermanence (i.e. everything comes and goes) (e.g. [37]). It has been demonstrated that mindfulness enables individuals to refrain from maladaptive impulsive behaviors [38]. Therefore, we conducted exploratory analyses to investigate whether the effect of IED on behavior is moderated by mindfulness skills.

In sum, the aim of the study is twofold. First, we investigated whether mindfulness is related to more congruent implicit and explicit attitudes and, if so, we explored which subskills are associated with that (RQ 1). Second, we assessed whether the mindfulness subskills moderate the relationship between IED and behavior (RQ 2a). Dual-process models, which postulate direct influences of implicit attitudes and explicit attitudes on behavior served as starting point for these two research questions. Socio-cognitive models, such as the Reasoned-Action Approach [39] or the Integrated-Change Model [40] state that the most important proximal determinant for behavior is intention. Currently, it is unclear whether IED affects behavior only or also its most proximate antecedent and whether this relationship might be moderated by the mindfulness subskills. Therefore, we additionally explored whether the mindfulness subskills moderate the relationship between IED and intention (RQ 2b).

The behavior chosen for this study is red meat consumption (RMC). RMC has been defined as a threat for people’s health (e.g. [41]) as well as for the environment [42, 43]. Moreover, former studies have shown that individuals, who consume meat can experience dissonance, e.g. by an inconsistency of cognitions (“I like to eat meat; I don’t like to hurt animals”) [44] or by an inconsistency between their behavior and their morale or values (conflict between enjoying meat and concern for animal welfare) [45], which is also described as “meat paradox” [46] in the literature. Also it was claimed that “Meat should be of special interest to psychologists, because it is a quintessential example of the interesting and important state of ambivalence” [47]. Based on this knowledge, we expected dissonance regarding RMC to exist between the implicit and the explicit level as it has been shown for other behaviors [28–31] and, therefore, selected the behavior at hand to answer the research questions. Moreover, shedding light on the relationships between IED and RMC could support the development of future interventions that are aiming to reduce RMC.

## Method

### Design

The current study is a follow-up study of a larger study, in which different predictive models of implicit attitudes and explicit cognitions regarding (the intention to reduce) red meat consumption (RMC) were tested. The study protocol of the original study was preregistered at https://osf.io/7enj9/?view_only=d1afaf26fdbe4f13a9feb0d857c89db0. IED and mindfulness were not part of the previous study. In the study at hand, emphasis lies on the relationships between mindfulness, IED, behavior, and intention.

### Ethical approval

The FHMLRec, the ethical committee of a Dutch University, provided ethical approval for this study (Muschalik/220517).

### Power analysis

To determine the sample size, we conducted a power analysis by means of G*Power. Small effect sizes were anticipated for main and interaction effects (f2 = 0.03) and the test power was set at 0.80 with a type I error rate of α = 0.05 for two-sided testing. The calculation revealed that a minimum sample of 488 is required. Based on former experiences of the internet research agencies that were collaborated with in this study, a drop-out of 60% between the baseline and the second follow-up (T2) was expected. Therefore, the aim was to have data of 1220 participants available at the first measurement (after having implemented various exclusions), in order to have data of 488 participants available at the second follow-up.

### Procedure

We conducted a three-wave longitudinal study with a baseline measurement (T0), a follow-up after one month (T1) and another follow-up after three months (T2). The whole study was conducted online among a sample of the Dutch population (47% female, age range: 18–89 years at baseline), which was recruited among members of two independent Internet panels that operate in line with the ISO standards [48, 49]. All individuals who were older than 18 years and had previously indicated to consume meat, were invited by email to participate.

When willing to participate, information was provided explaining that the study aims to gain insight into the relationship between determinants related to eating behavior and that there would be three measurements, that one measurement would take 15–20 min to complete, that each measurement was comprised of a reaction time task and a questionnaire, that participation was free of risks, that all data would be gathered and analyzed anonymously, and that they would receive a monetary reward. To begin with the study, participants needed to read and agree upon an electronic informed consent. If participants did not do so, they were excluded from further participation. At the beginning of the study, we included a question to double-check whether only people who consumed red meat at least once a month participated. People who answered the question with “no” were excluded. One and three months after baseline, a new invitation was sent to those participants who had participated previously to complete the follow-up. Depending on the standards for payment used in the two different Internet panels, participants received €2.50 or €4.75 for participation in the baseline, €2.50 or €3.00 for participation in T1, and €4.00 or €5.00 for participation in T2.

### Measurements

#### Implicit attitude assessment task

The Single-Category Implicit Association Test (SC-IAT; [50]) was used to assess participants’ implicit attitudes towards red meat. In earlier studies, the SC-IAT has demonstrated satisfactory internal consistency [50]. Opposed the IAT, the SC-IAT does not require a contrasting concept (e.g. man vs. women) but measures the implicit attitude unrelated to a contrast category. As we were interested in the implicit attitudes towards red meat unrelated to a contrast category, the SC-IAT was selected. Positive and negative words from the Affective Norms for English Words (ANEW [51];) were used as evaluative stimuli. They were translated forth and back from English to Dutch by Dutch native researchers of Maastricht University. Subsequently, the Dutch words were pretested regarding their perceived levels of valence (1 = ‘very negative’ to 9 = ‘very positive’), arousal (1 = ‘not arousing at all’ to 9 = ‘very arousing’), and familiarity (1 = ‘very unfamiliar’ to 9 = ‘very familiar’) by 28 people. Words with the highest scores regarding positivity and familiarity and similar arousal levels were selected as positive stimuli (*love*, *friend*, *freedom*, *humor*, *joy;* translated from Dutch). Words with the lowest scores on positivity, highest scores of familiarity and similar evaluations of arousal were selected as negative stimuli (*death*, *hate*, *devil*, *loneliness*, *lie*; translated from Dutch). To represent red meat, pictures were derived from the study of De Houwer and De Bruycker [52] and from the Internet (i.e., Creative Commons Images). These were pretested by the same sample regarding their representativeness for red meat (1 = ‘not representative at all’, 2 = ‘not so strongly/a bit representative’, 3 = ‘strongly representative’). Seven pictures that were evaluated as to be the most representative, were included in the SC-IAT.

We programmed the SC-IAT by means of the software Inquisit by Millisecond (Version 4) with the script being based on Karpinski and Steinman [50]. The SC-IAT is comprised of two blocks which each consisted of 24 practice trials and 72 test trials. In one block “red meat or negative” versus “positive” are the two categories, in the reversed block “red meat or positive” versus “negative” built the two categories. One after another, negative or positive words or pictures of red meat were presented in the middle of the screen and participants were instructed to indicate as quickly as possible to which of the two categories the stimulus belonged. The two blocks were presented in a counterbalanced order. The assumption that underlies the SC-IAT is that when an individual is quicker with categorizing the stimuli when “red meat or negative” are one category than when “red meat or positive” are one, the individual’s implicit attitude regarding red meat is negative and vice versa. Throughout the whole task, labels of the categories were displayed on the right and left upper part of the computer screen. When a stimulus belonged to the category that was shown on the right upper part of the screen, participants had to press *i* on their keyboard. When the stimulus belonged to the category displayed on the left upper part of the screen, they had to press *e*. All stimuli were presented equally frequent and randomized. If an answer was incorrect, a red X appeared on the screen until it was corrected.

The implicit attitude was represented by d-scores, which were calculated by the Inquisit software. The d-score represents the strength of an association between concepts, which is measured by the standardized mean difference score of the ‘hypothesis-inconsistent’ pairings and ‘hypothesis-consistent’ pairings and is expressed in milliseconds [53]. More positive d-scores indicate a more positive reaction to red meat. Normally, d-scores range from − 2 to 2 and all participants in our sample had a d-score within this range. After they had performed the SC-IAT, participants were asked whether they were distracted during the task. Different types of distraction were offered (e.g. ‘I was talking on the phone’, ‘I was eating or drinking’, ‘I was listening to music’ etc.). Only when participants selected the option ‘I was not distracted’, their d-score was included in the analyses. Based on this control question, data of 185 participants (13%) were excluded afterward. The internal reliability of the SC-IAT was calculated by dividing the SC-IAT into thirds (blocks of 24 test trials). For each third, a SC-IAT was calculated [50] and the average intercorrelation among these three scores was identified by means of the Spearman-Brown formula, which is conceptually equivalent and comparable to Cronbach’s alpha in terms of range (i.e., from 0 to 1, where higher values indicate stronger internal consistency). The internal consistency was deemed acceptable (r = .73). The test-retest correlations between implicit attitudes at baseline and T1 (r =. 18, *p* < .01), implicit attitude at baseline and T2 (r = .09, *p* < .05), and implicit attitude at T1 and T2 (r =. 21, p < .01) were significant, but fall within the lower range of test-retest reliabilities. However, these values are comparable to published studies using implicit measures such as a race-attitude Implicit Association Tests (0.17–0.50) [54], aggressiveness Implicit Association Tests (0.14–0.39) [55] or political Single Target-Implicit Association Tests (0.21–0.46) [56].

Subsequently, participants filled in a questionnaire, which is described in the following. The SC-IAT was performed first, as a prior assessment of explicit cognitions is assumed to trigger red meat-related thoughts which would in turn influence the reaction time in a following task [57].

#### Self-report assessment

The I-Change model [40, 58] has previously been used to identify eating-related cognitions [59, 60] and was used in the present study to assess explicit attitudes towards RMC and intention to reduce RMC. The questionnaire can be found at https://osf.io/bkp5r/?view_only=6c2e208b9e8f4354ac339c2596b85c2f.

*Explicit attitude* was assessed with the two scales perceived pros and perceived cons regarding RMC. Each scale is comprised of 10 statements on a 5-point Likert Scale, which were based on beliefs underlying the attitudes towards meat [61, 62]. An example of a pro statement is “Eating red meat is” (1) “very nutritious” to (5) “not nutritious”. As two items showed a low factor loading, they were removed from the scale (Ω = .73). Items were reversed so that higher values represent the perception of more pros. An example of a con statement is “Eating red meat is” (1) “very disgusting” to (5) “not disgusting”. Due to a low factor loading one item was removed (Ω = .66). For the analysis, a sum score for the pro scale and a sum score for the con scale was created. Both scales were added to represent one scale for explicit attitude (range − 40 to 40) that was used in the analyses. The higher the score, the more positive the explicit attitude.

*Intention* to reduce RMC was measured by three different items. The first item, labeled *intention planning*, asked “Are you planning to eat less red meat in the future?” with answer options ranging from (1) “No, I am not planning to reduce my red meat intake” to (7) “Yes, within one month”. The second item, labeled *intention likeliness*, asked “The chance that I will eat less red meat within the next three months is” (1) “very unlikely” to (5) “very likely”. The third item, labeled *intention strength*, asked participants to indicate on how strongly he/she was planning to reduce his/her red meat intake within the next three months with a scale from (1) “very little” to (9) “very strongly”. Factor saturation of the standardized sum scores was estimated as insufficient (Ω = .07), therefore intention items were entered separately in the analyses as. Higher scores on all items represent a stronger intention to reduce RMC.

To assess *mindfulness*, a Dutch translation of the KIMS-E [17, 18] was administered. In contrast to earlier studies that suggested mindfulness to be a unidimensional construct [63, 64], a more recent factor analysis regarding various mindfulness questionnaires identified five different domains of trait mindfulness [17]. Given that the original KIMS [25] only consists of four facets, we decided to use the KIMS-E, which entails the fifth facet. Also, the KIMS-E has demonstrated good psychometric properties [18]. The KIMS-E is a 46-item scale which entails the mindfulness subskills observing, describing, acting with awareness, and accept without judgment as well as the additional subskill non-reactivity to inner experience derived from the Five-Factor Mindfulness Questionnaire [17]. All items were rated on a 5-point Likert scale ranging from (1) “never or very rarely true” to (5) “always or almost always true”. Where appropriate, items were reversed so that higher scores indicate higher levels of mindfulness.

*Observing* was assessed by means of 12 items. An example is “I notice changes in my body, such as whether my breathing slows down or speeds up.” Due to low factor loadings, two items were removed. A mean scale score of all remaining items was included in the analyses (Ω = .66).

*Describing* was measured by eight items, such as “Even when I’m feeling terribly upset, I can find a way to put it into words.” We included a mean scale score for describing (Ω = .80) in the analyses.

*Acting with awareness* was administered by ten items. An example is “When I’m doing something, I’m only focused on what I’m doing, nothing else.” Due to low factor loadings, four items were removed and a mean scale score was created out of the remaining items and included in the analyses (Ω = .56).

*Accept without judgment* was comprised of nine items. Example items are “I criticize myself for having irrational or inappropriate emotions” or, “I think some of my emotions are bad or inappropriate and I shouldn’t feel them.”. One item showed a low factor loading and was removed. All other items were combined to a mean scale score, which was included in the analyses (Ω = .83).

*Nonreactivity to inner experience* was assessed by seven items. Example items are “Usually when I have distressing thoughts or images, I just notice them and let them go” or “I perceive my feelings and emotions, without having to react to them”. Due to low factor loadings, three items were removed and a mean scale score of the remaining items was included in the analyses (Ω = .70). As mindfulness has been shown to be a multidimensional and not a unidimensional construct [17] and since we were interested in the specific sub-skill(s) that might be associated with attitudinal dissonance, the sub-skills were entered as separate constructs in the analyses.

*Red meat consumption* was measured by means of the question “On how many days per week do you usually consume red meat” (ranging from 1 to 7 days per week and the additional answer option ‘Not on a daily basis but at least once a month’) and the open question “On days when you eat red meat, how many grams of red meat do you eat on average per day?” A reference point was provided, e.g. that a piece of prepared meat at the main meal equals 100 g and a slice of meat topping (e.g. ham) equals 15 g. By multiplying the frequency by the number of grams, the weekly RMC was calculated. This procedure was based on the Food Frequency Questionnaire (FFQ) and former diet-related studies [65, 66].

Two control questions were formulated (e.g. ‘From the following answer options, please select statement 4’) to excluded data of those participants who did not answer the questionnaire thoroughly (e.g., straightlining or not paying attention).

### Analyses

Scale quality of the measurements used in the present study was assessed by means of exploratory factor analyses as well as McDonald’s omega (reported in the measurement section above), which is considered to be less biased than Cronbach’s alpha [67]. Omega_hierarchical_ was used as an indicator of internal structure, which estimates factor saturation based upon the sum of the squared loadings of items on the general factor [68].

Logistic regressions were used to investigate whether dropout at T1 and T2 was predicted by the variables age, gender, educational level, explicit attitude, implicit attitude, IED, intention, RMC, and the mindfulness subskills. To assess whether the measured variables differed over time, we conducted analyses of variance (ANOVAs).

Based on previous studies regarding IED [29, 69, 70], we created an index to analyze the effects of IED. The index was formed by calculating the absolute value of the difference between the average of a participant’s standardized reaction time of the SC-IAT and the standardized explicit attitude score. This index shows where each participant falls within the distribution of the sample on the implicit measure versus the explicit measure. When the index shows a value close to zero, a person’s place in the distribution is similar for the implicit and explicit measure (e.g. high in the distribution of both measures, low in the distribution of both measures, and so on). The more the score on the index is away from zero, the more the person’s two attitudes deviate from each other (e.g. high in the distribution of explicit attitudes and low in the distribution of implicit attitudes or vice versa). Thereby, the size of the discrepancy is indicated.

To assess possible cross-sectional and longitudinal associations between IED and the mindfulness subskills (RQ 1), we conducted correlational analyses between the baseline mindfulness subskills and IED at baseline, after one month, and after three months.

To assess a possible moderating effect of the mindfulness subskills on the relationship between IED and RMC (RQ 2a), three regressions were conducted. For short-term effects, we regressed participant’s RMC levels at T0 on T0 IED and the mindfulness subskills observing, describing, acting with awareness, accepting without judgment, and nonreactivity in step one, and added the interactions between IED and all five mindfulness subskills in a second step. To assess long-term effects, the same regressions were repeated but with RMC at T1 and T2 as the dependent variable.

To investigate the additional question regarding intention and possible short-term and long-term effects of the mindfulness subskills on the relationship between IED and intention (RQ 2b), we conducted three regressions each with intention at baseline, at T1, and at T2 as the dependent variable. Baseline variables were again added in two steps of a regression. IED and the mindfulness subskills in step one, and the interactions between IED and the mindfulness subskills in a second step. In case significant interaction terms were found, follow-up stratified analyses were conducted [71]. In this case, the respective subskill was categorized into low, moderate, and high based on one standard deviation below, on, and above the mean.

To control for multiple testing, we used the Benjamini-Hochberg [72, 73] linear step-up method for the regression models. This method is considered more powerful and less conservative than the Bonferroni procedure [72]. By means of an Excel template, the adjusted *p*-values were calculated [74]. The Benjamini-Hochberg method ranks variables according to their p-values in increasing order. The smallest value gets rank 1, the second rank 2, and the largest value receives rank N. Then, each p-value is multiplied by N and divided by its assigned rank to give the adjusted Benjamini- Hochberg p-value. For all regressions and stratified analyses, Benjamini-Hochberg p-values are reported, with a false discovery rate at 25%.

## Results

At baseline, 1790 individuals participated. Participants who indicated that they were distracted during the SC-IAT or did not answer the control questions correctly were removed. Thereby a sample of 1476 remained (47% female, mean age = 49, SD = 15.90). After one month, data of 980 participants were available, out of which 272 were excluded for the same reasons as mentioned above. Thus, the sample at T1 consisted of 708 participants (48% of baseline, 47% female, mean age = 48, SD = 15.18). At T2, data of 556 participants were available out of which 89 were excluded. The remaining sample at T2 consisted of 467 data (32% of baseline, 44% female, mean age = 50, SD = 15.67). Drop-out at T1 was significantly predicted by the mindfulness subskill acceptance without judgment (OR = 1.34, 95%CI [1.13, 1.58], *p* < .001) indicating that a higher acceptance was associated with a higher likeliness to drop-out. This variable was added to all analyses. Drop-out at T2 was not predicted by any of the measured variables.

**Fig. 1:**
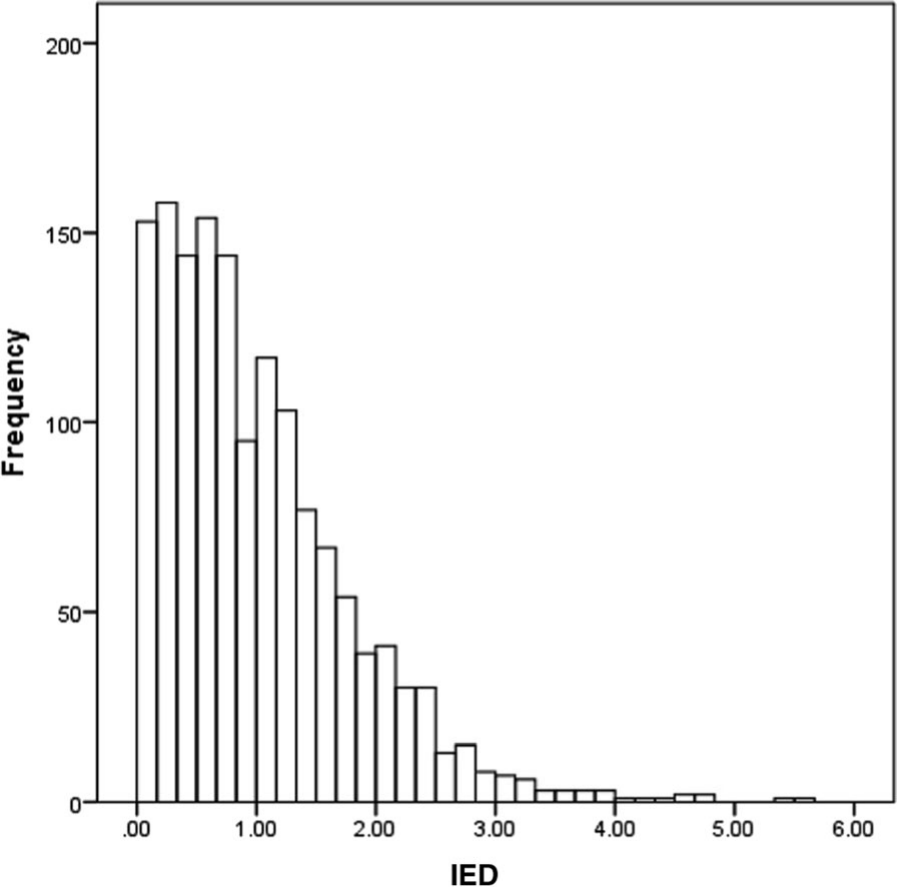
Distribution of IED at baseline

The implicit attitude towards red meat of the sample was slightly negative (M = −.03, SD = .32) and the explicit attitude was slightly positive (M = 9.53, SD = 8.25). Implicit and explicit attitude were significantly positively correlated with each other (r = .17). IED was present and moderately distinct (M = 1.01, SD = .80, range .00–5.51) and negatively correlated with implicit and explicit attitude (r = −.06, r = −.18). The distribution of IED at baseline is presented in Fig. 1. Descriptives of all study variables and changes over time are displayed in Table 1. The mindfulness subskills describing, acting with awareness, and acceptance without judgment changed significantly over time (i.e. decreased over time).

**Table 1:** Descriptives and differences of study variables over time

	T0 (*N* = 1476)	T1 (*N* = 708)	T2 (*N* = 467)	F	df	p
	M (SD)	M (SD)	M (SD)	F	df	p
Explicit attitude	9.53 (8.25)	9.57 (8.36)	9.35 (8.57)	.10	2	.90
Implicit attitude	−.03 (.32)	−.06 (.32)	−.05 (.31)	2.83	2	.06
IED	1.01 (.80)	1.03 (.79)	1.05 (.76)	.68	2	.50
Observing	3.26 (.58)	3.21 (.62)	3.24 (.63)	1.22	2	.30
Describing	3.58 (.65)	3.01 (.33)	3.00 (.35)	376.36	2	< .001
Acting with awareness	3.21 (.59)	2.87 (.49)	2.87 (.48)	123.94	2	< .001
Accepting without judgment	3.76 (.71)	2.15 (.74)	2.20 (.74)	1567.98	2	< .001
Non-reactivity	3.06 (.67)	3.01 (.67)	3.13 (.66)	1.61	2	.20
Intention (item 1)	2.24 (1.93)	2.30 (1.99)	2.40 (2.02)	1.26	2	.29
Intention (item 2)	2.08 (1.10)	2.03 (1.08)	2.11 (1.08)	.81	2	.45
Intention (item 3)	3.49 (2.53)	3.48 (2.53)	3.67 (2.54)	1.09	2	.34
Red meat consumption (gr/week)	473.50 (435.77)	493.06 (388.34)	484.23 (344.78)	.57	2	.57

### RQ1 Is mindfulness associated with congruent implicit and explicit attitudes?

Correlations between study variables at baseline as well as correlations between IED at T1 and T2 with mindfulness subskills are depicted in Table 2. Contrary to our expectation, none of the mindfulness subskills were associated with IED at baseline nor with IED after one or three months.

**Table 2:** Correlations (and 95% CI) between study variables at baseline and IED at T1 and T2 with mindfulness subskills

	1	2	3	4	5	6	7	8	9	10	11	12	13
1. Implicit attitude													
2. Explicit attitude	.17** (.12-.22)												
3. RMC	.09** (.04-.14)	.29** (.24-.34)											
4. Intention planning	-.06* (-.11- -.01)	-.43** (-.47- -.39)	-.11** (-.16- -.06)										
5. Intention likeliness	-.08** (-.13- -.03)	-.49** (-.53- -.45)	-.17** (-.22- -.12)	.71** (.68-.73)									
6. Intention strength	-.07** (-.12- -.02)	-.46** (-.50- -.42)	-.19** (-.24- -.14)	.70** (.67-.72)	.82** (.08-.84)								
7. Observing	-.09** (-.14- -.04)	-.11** (-.16- -.06)	-.09** (-.14- -.04)	.12** (.07-.17)	.14** (.09-.19)	.17** (.12-.22)							
8. Describing	-.06* (-.11- -.01)	.08** (.03-.13)	-.02 (-.07-03)	.00 (-.05-.05)	-.01 (-.06-.04)	-.01 (-.06-.04)	.36** (.31-.40)						
9. Acting with awareness	.03 (-.08-.02)	.18** (.13-.23)	.06* (.01-.11)	-.08** (-.13- -.03)	-.10** (-.15-.05)	-.07** (-.12-.02)	-.06* (-.11- -.01)	.21** (.16-.26)					
10. Acceptance without judgment	.01 (-.04-.06)	.14** (.09-.19)	.06* (.01-.11)	-.09** (-.14- -.04)	-.11** (-.16- -.06)	-.14** (-.19-.09)	-.20** (-.25- -.15)	.23** (.18-.28)	.36** (.31-.04)				
11. Nonreactivity	.03 (-.08-.02)	.07** (.02-.12)	.03 (-.08-.02)	-.04 (-.09-.01)	-.03 (-.08-.02)	-.01 (-.06-.04)	.23** (.18-.28)	.29** (.24-.34)	.16** (.11-.21)	.15** (.10-.20)			
12. IED (T0)	-.06* (-.11- -.01)	-.18** (-.23- -.13)	-.09** (-.14- -.04)	.11** (.06-.16)	.11** (.06-.16)	.10** (.05-.15)	.05 (.00-.10)	.02 (-.03-.07)	.03 (-.02-.08)	.03 (-.02-.08)	.03 (-.02-.08)		
13. IED (T1)							.06 (-.01-.13)	.01 (-.06-.08)	-.01 (-.08-.06)	-.03 (-.10-.04)	-.03 (-.10-.04)	.19** (.12-.26)	
14. IED (T2)							-.03 (-.12-.06)	.00 (-.09-.09)	-.03 (-.12-.06)	.00 (-.09-.09)	-.09 (-.18-.00)	.14** (.05-.23)	.25** (.16-.33)

** *p* < .01, * *p* < .05

### RQ2a Do the mindfulness subskills moderate the relationship between IED and RMC?

#### RMC at baseline

For RMC at baseline, no significant interactions between IED and the mindfulness subskills were found. This indicates that, contrary to our expectation, mindfulness subskills do not moderate the relationship between IED and RMC. Tests for multicollinearity indicated a very low level of multicollinearity between the mindfulness subskills (*VIF* = 1.33 for observing, 1.36 for describing, 1.21 for acting with awareness, 1.30 for acceptance without judgment, and 1.15 for nonreactivity). Thus, nonsignificant findings are not due to multicollinearity. Lower RMC at baseline was significantly associated with higher IED (B = − 49.09, *p* = .004, 95%CI [− 76.69, − 21.49]), a higher distinction of the subskill observing (B = − 63.45, p = .004, 95%CI [− 106.91, − 20.00]), and a lower distinction of the subskills acting with awareness (B = 32.77, *p* = .23, 95%CI [− 7.79, 73.32]) and nonreactivity (B = 31.31, *p* = .22, 95%CI [− 4.00, 66.62]) (see Table 3 for all regression coefficients).

**Table 3:** Coefficients of the multiple regression analyses with RMC at T0, T1, and T2 as dependent variable. Interactions with IED are added at step 2

		RMC								
		T0		T1			T2			
Step	Independent variables	B	95% CI	p^a^	B	95% CI	p^a^	B	95% CI	p^a^
1	IED	− 49.09	− 76.69- -21.49	.004^b^	− 44.23	− 79.77- -8.69	.05 ^b^	−38.34	−75.51- -1.16	.09 ^b^
	Observing	−63.45	−106.91- -19.99	.004 ^b^	−77.61	− 131.57- -23.65	.008 ^b^	− 79.90	− 143.32- -16.48	.09 ^b^
	Describing	−7.93	−47.72-31.86	.74	−14.91	−67.23-37.41	.71	−3.00	−64.02-58.03	.92
	Acting with awareness	32.77	−7.79-73.32	.23 ^b^	50.85	−1.58-103.27	.19 ^b^	35.07	−26.56-96.71	.37
	Accepting without judgment	15.46	−19.88-50.80	.60	22.47	−21.86-66.80	.66	29.31	−21.15-79.78	.37
	Nonreactivity	31.31	−4.00-66.62	.22 ^b^	15.60	−29.44-60.65	.71	39.95	−10.12-90.01	.28
2	IED	−52.65	−80.45--24.85	.004 ^b^	−46.58	−82.50--10.66	.05 ^b^	−36.62	−74.83-1.59	.23
	Observing	−65.43	−108.88- -21.97	.004 ^b^	−79.09	− 133.31- -24.88	.008 ^b^	− 78.83	− 142.82- -14.83	.11 ^b^
	Describing	−9.36	−49.12-30.40	.73	−16.43	−69.06-36.19	.71	−4.09	−65.30-57.13	.92
	Acting with awareness	36.51	−4.25- 77.27	.22 ^b^	54.50	1.69–107.31	.16 ^b^	38.52	−23.72-100.76	.37
	Accepting without judgment	12.50	−22.84- 47.84	.64	19.31	−25.22-63.84	.69	29.77	−21.10-80.63	.37
	Nonreactivity	30.37	−4.90-65.65	.22 ^b^	15.92	−29.28-61.11	.71	38.67	−11.55-88.90	.28
	IED x observing	−8.86	−63.66-45.95	.75	−6.00	−76.31-64.31	.87	7.25	−69.56-84.06	.92
	IED x describing	18.77	−30.57-68.12	.64	−7.00	− 73.59-59.59	.87	− 31.87	− 104.72-40.99	.51
	IED x acting with awareness	40.88	−12.87-94.63	.26	42.35	−24.71-109.40	.59	21.80	−56.12-99.72	.70
	IED x accepting without judgment	13.43	−31.86-58.71	.68	10.75	−44.88-66.38	.80	−46.55	−107.05-13.95	.28
	IED x nonreactivity	24.88	−16.58-66.35	.41	30.49	−31.06-92.03	.66	53.48	−11.87-118.83	.11

^a^*p*-values after correction for multiple testing according to Benjamini-Hochberg

^b^ significant *p*-values after correction for multiple testing according to Benjamini-Hochberg

#### RMC after one month

For RMC after one month, no significant interactions between IED and the mindfulness subskills were found. Again, multicollinearity was very low (*VIF* = 1.32 for observing, 1.41 for describing, 1.24 for acting with awareness, 1.27 for acceptance without judgment, and 1.17 for nonreactivity) and can be excluded as a possible reason for nonsignificant findings. Lower RMC after one month was associated with higher IED (B = − 44.23, *p* = .05, 95%CI [− 79.77, − 8.69]), a more distinct observing subskill (B = − 77.61, *p* = .008, 95%CI [− 131.57, − 23.65]), and with a lower distinct subskill acting with awareness (B = 50.85, *p* = .19, 95%CI [− 1.58, 103.27]) (see Table 3 for all regression coefficients).

#### RMC after three months

Also for RMC after three months, no significant interactions between IED and the mindfulness subskills were found. Multicollinearity was again very low (*VIF* = 1.35 for observing, 1.52 for describing, 1.29 for acting with awareness, 1.44 for acceptance without judgment, and 1.17 for nonreactivity) and can, therefore, be ruled out as possible reason for nonsignificant findings. After three months, lower RMC was associated with higher IED (B = − 38.34, *p* = .09, 95%CI [− 75.51, − 1.16]) and with a more distinct observing subskill (B = − 79.90, p = .09, 95%CI [− 143.32, − 16.48]) (see Table 3 for all regression coefficients).

### RQ2b Do the mindfulness subskills moderate the relationship between IED and the intention to reduce RMC?

#### Intention at baseline

For the items *intention planning* and *intention strength*, a significant interaction between IED and accepting without judgment was found at baseline (intention planning: B = −.20, *p* = .11, 95%CI [−.40, −.001]; intention strength: B = −.30, *p* = .05, 95%CI [−.56, −.04]) (see Table 4).

**Table 4:** Coefficients of the multiple regression analyses with intention items at T0, T1, and T2 as dependent variable. Interactions with IED are added at step 2

		T0									T1									T2								
		Intention planning			Intention likeliness			Intention strength			Intention planning			Intention likeliness		Intention strength				Intention planning		Intention likeliness			Intention strength			
Step	Independent variables	B	95% CI	p ^a^	B	95% CI	p ^a^	B	95% CI	p ^a^	B	95% CI	p ^a^	B	95% CI	p ^a^	B	95% CI	p ^a^	B	95% CI	p ^a^	B	95% CI	p ^a^	B	95% CI	p ^a^
1	IED	.26	.14-. 38	.004 ^b^	.15	.08–.22	.004 ^b^	.32	.16-. 47	.003 ^b^	.26	.08–.44	.004 ^b^	.17	.07–.27	.009 ^b^	.43	.20–.66	.009 ^b^	.29	.07–.51	.09^b^	.16	.04–.27	.09 ^b^	.36	.09–.64	.09 ^b^
	Observing	.40	.21–.59	.004 ^b^	.26	.16–.37	.004 ^b^	.70	.45-. 95	.003 ^b^	.41	.14–.69	.004 ^b^	.12	−.03–.27	.21 ^b^	.40	.05–.75	.10 ^b^	.39	.02–.76	.09 ^b^	.11	−.09–.31	.61	.40	−.07–.87	.31
	Describing	−.03	−.21–.14	.85	−.04	−.14–.06	.56	−.16	−.39–.06	.34	.11	−.15–.38	.53	.04	−.11–.18	.79	.03	−.31–.37	.97	.42	.06–.77	.09 ^b^	.09	−.10–.28	.61	.14	−.31–.59	.90
	Acting with awareness	−.18	−.36–.00	.11 ^b^	−.12	−.22--.02	.06 ^b^	−.07	−.30–.16	.61	−.23	−.49–.04	.19 ^b^	−.14	−.29–.00	.17 ^b^	−.27	−.61–.07	.23 ^b^	−.07	−.43–.29	.86	−.17	−.36–.03	.28	−.02	−.47–.44	.96
	Accepting without judgment	−.11	−.26–.05	.28	−.08	−.17–.01	.17 ^b^	−.31	−.52--.11	.003 ^b^	−.09	−.31–.14	.53	−.13	−.25–.00	.17 ^b^	−.30	−.59 - -.02	.11 ^b^	−.09	−.39–.20	.81	−.14	−.30–.02	.28	−.41	−.79- -.04	.13 ^b^
	Nonreactivity	−.15	−.30–.01	.11 ^b^	−.07	−.15–.02	.24 ^b^	−.09	−.29–.11	.60	−.21	−.44–.02	.19 ^b^	−.03	−.16–.09	.79	−.15	−.45–.14	.40	−.34	−.63–.05	.09 ^b^	.01	−.15–.16	.93	.01	−.36–.38	.96
2	IED	.28	.15-. 40	.004 ^b^	.16	.09–.23	.004 ^b^	.33	.17-. 49	.003 ^b^	.29	.10–.47	.004 ^b^	.19	.09–.29	.009 ^b^	.48	.24–.71	.009 ^b^	.32	.09–.54	.09 ^b^	.15	.03–.27	.09 ^b^	.36	.08–.65	.09 ^b^
	Observing	.41	.22-. 60	.004 ^b^	.27	.16–.38	.004 ^b^	.70	.45-. 94	.003 ^b^	.40	.13–.68	.004 ^b^	.12	−.03–.27	.21 ^b^	.39	.04–.74	.10 ^b^	.39	.01–.76	.09 ^b^	.11	−.10–.31	.61	.36	−.12–.83	.40
	Describing	−.02	−.20–.15	.85	−.04	−.14–.06	.57	−.15	−.38–.07	.34	.10	−.17–.37	.53	.02	−.12–.17	.79	−.01	−.35–.33	.97	.40	.04–.76	.09 ^b^	.09	−.10–.29	.61	.13	−.33–.58	.90
	Acting with awareness	−.19	−.37--.01	.11 ^b^	−.13	−.23--.03	.03 ^b^	−.08	−.31–.15	.61	−.22	−.49–.04	.19 ^b^	−.13	−.28–.01	.17 ^b^	−.28	−.62–.07	.23 ^b^	−.08	−.45–.29	.86	−.17	−.37–.03	.28	−.05	−.51–.42	.96
	Accepting without judgment	−.10	−.25–.06	.31	−.08	−.17–.01	.17 ^b^	−.31	−.51--.11	.003 ^b^	−.08	−.30–.15	.50	−.12	−.24–.01	.17 ^b^	−.28	−.57–.01	.15 ^b^	−.07	−.37–.23	.86	−.14	−.30–.02	.28	−.41	−.78- -.03	.13 ^b^
	Nonreactivity	−.15	−.30–.01	.11 ^b^	−.07	−.15–.02	.24 ^b^	−.09	−.29–.11	.60	−.20	−.43–.03	.19 ^b^	−.03	−.15–.10	.79	−.15	−.44–.15	.40	−.34	−.63 - -.04	.03	.01	−.15–.17	.93	.02	−.35–.39	.96
	IED x observing	−.03	−.27–.21	.85	−.02	−.16–.12	.81	.03	−.28–.35	.84	.05	−.31–.41	.79	.00	−.20–.19	.97	.24	−.21–.70	.40	.04	−.41–.49	.91	.04	−.20–.29	.90	.37	−.20–.94	.49
	IED x describing	.02	−.20–.23	.88	.01	−.11–.14	.83	.10	−.18–.38	.61	.19	−.15–.53	.42	.09	−.09–.27	.56	.27	−.16–.70	.36	.16	−.27–.59	.78	.05	−.18–.28	.90	.19	−.35–.73	.90
	IED x acting with awareness	−.17	−.40–.07	.28	−.09	−.22–.05	.31	−.09	−.40–.22	.61	.12	−.22–.46	.53	.04	−.15–.23	.79	.02	−.41–.45	.97	−.19	−.64–.27	.78	.02	−.23–.26	.93	−.15	−.73–.43	.90
	IED x accepting without judgment	−.20	−.40–.00	.11 ^b^	−.06	−.17–.06	.44	−.30	−.56--.04	.05 ^b^	−.28	−.57–.00	.17 ^b^	−.23	−.38 - -.07	.02 ^b^	−.50	−.85 - -.14	.06 ^b^	−.04	−.39–.32	.91	.05	−.14–.24	.90	.19	−.26–.64	.90
	IED x nonreactivity	.09	−.09–.27	.45	.04	−.07–.14	.58	.09	−.15–.32	.61	−.23	−.54–.08	.26	−.03	−.20–.14	.79	−.22	−.62–.17	.40	−.01	−.40–.37	.95	−.04	−.25–.17	.90	−.11	−.60–.37	.65

*Note.* B = unstandardised regression coefficient

^a^*p*-values after correction for multiple testing according to Benjamini-Hochberg

^b^ significant p-values after correction for multiple testing according to Benjamini-Hochberg

**Fig. 2:**
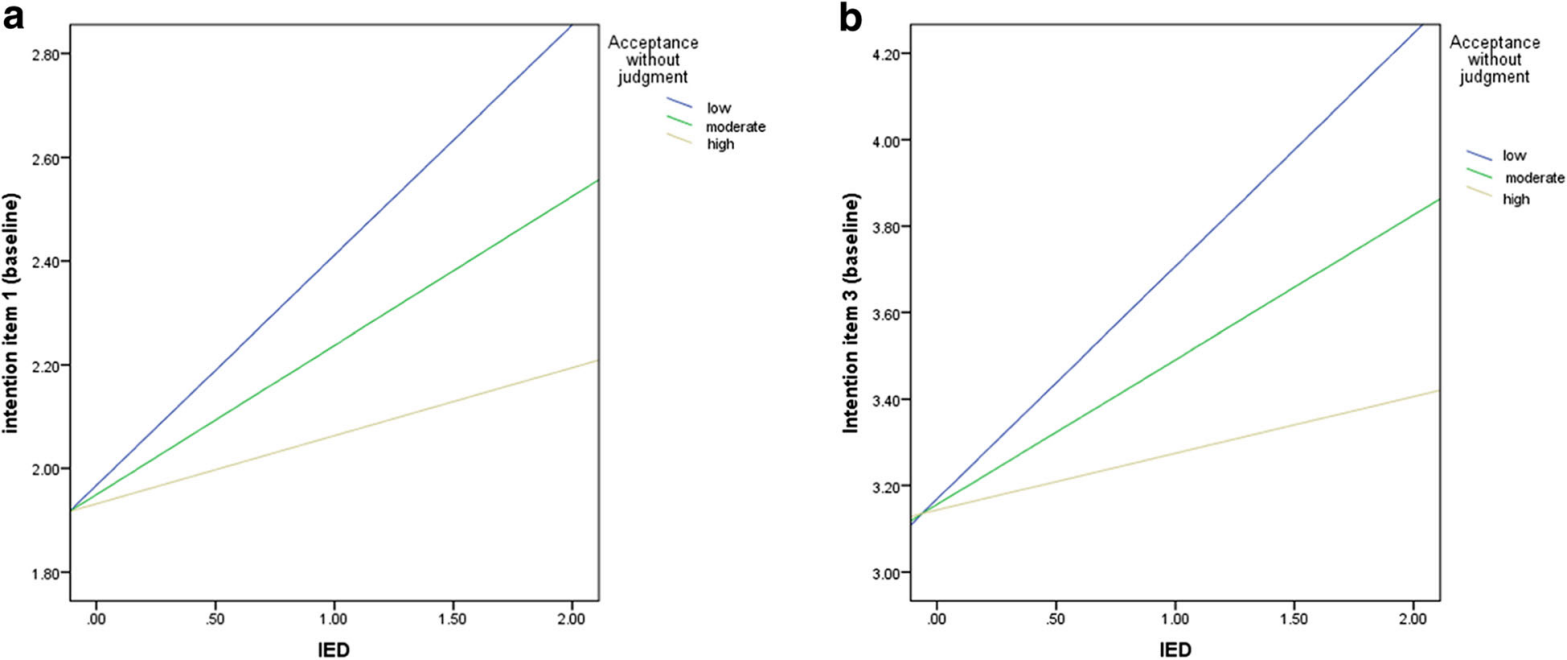
**a**. Stratified analyses for the relationship between intention (item 1) and IED and the moderator acceptance without judgment. **b**. Stratified analyses for the relationship between intention (item 3) and IED and the moderator acceptance without judgment

Stratified analyses for *intention planning* revealed that IED had a positive effect on the intention to reduce RMC when accepting without judgment was low (B = .43, *p* = .002, 95%CI [.26, .61]) and moderate (B = .28, p = .002, 95%CI [.16, .40]), but not when it was high (B = .12, *p* = .14, 95%CI [−.04, .28]) (see Fig. 2a).

Stratified analyses for *intention planning* revealed similar results. IED had a positive effect on the intention to reduce RMC when the ability to accept without judgment was low (B = .54, p = .002, 95%CI [.31, .77]) or moderate (B = .34, p = .002, 95%CI [.18, .50]), but not when it was high (B = .13, *p* = .22, 95%CI [−.08, .34]) (see Fig. 2b).

For *intention likeliness*, no significant interaction was found. In all three regressions, multicollinearity between the mindfulness subskills was very low (*VIFs* < 1.42). Hence, nonsignificant findings for this item are not due to multicollinearity.

#### Intention after one month

For all three intention items after one month, the interaction between IED and accepting without judgment was significant (item 1: B = −.28, *p* = .17, 95%CI [−.57, .002]; item 2: B = −.23, *p* = .02, 95%CI [−.38, −.07]; item 3: B = −.50, *p* = .06, 95%CI [−.85, −.14]) (see Table 4).

**Fig. 3:**
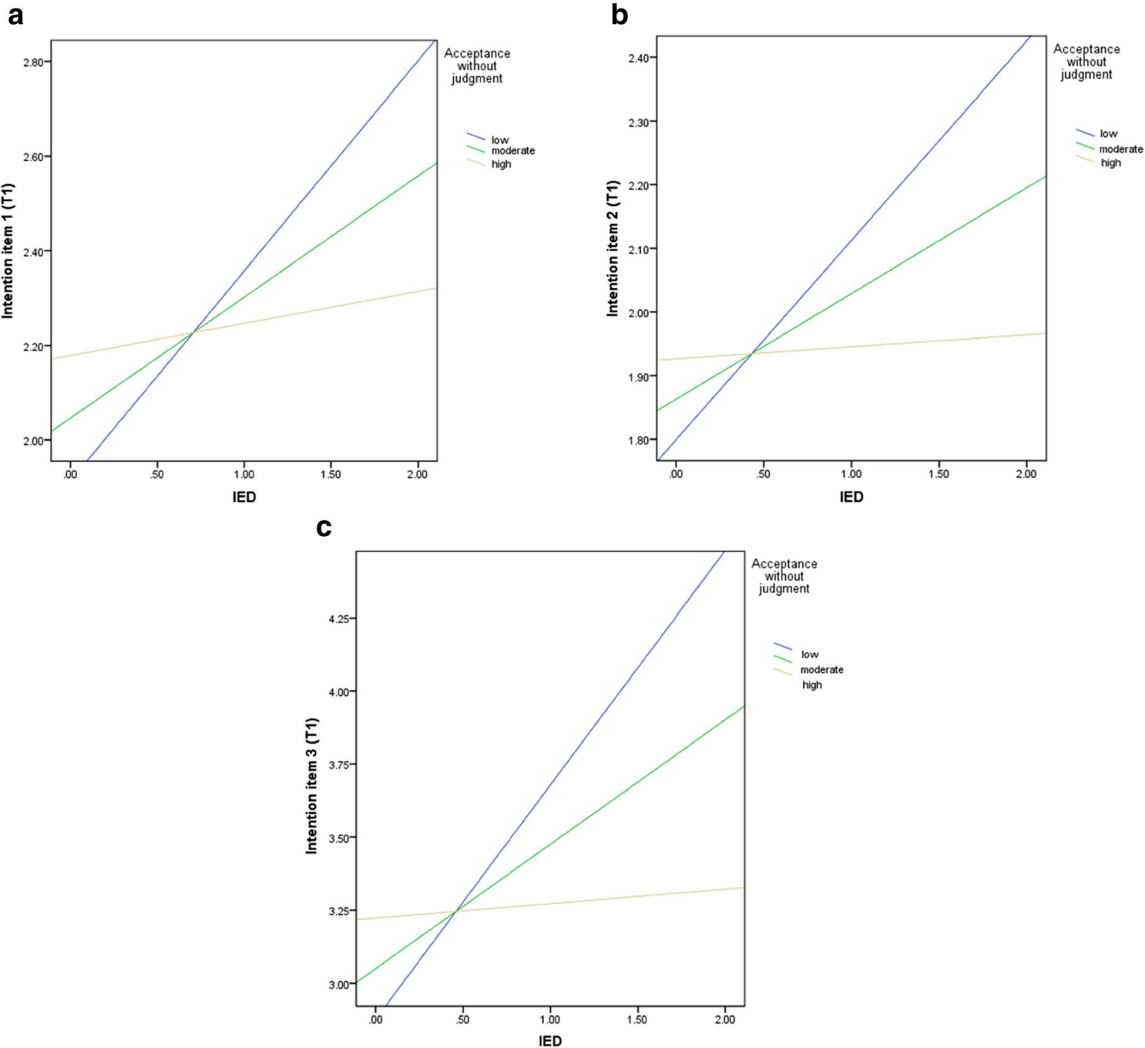
**a**. Stratified analyses for the relationship between. Intention (item 1) and IED and the moderator acceptance without judgment. **b**. Stratified analyses for the relationship between intention (item 2) and IED and the moderator acceptance without judgment. **c**. Stratified analyses for the relationship between intention (item 3) and IED and the moderator acceptance without judgment

Stratified analyses for *intention planning* showed that IED had a positive effect on the intention to reduce RMC when accepting without judgment was low (B = .44, *p* = .003, 95%CI [.19, .70]) and moderate (B = .26, *p* = .009, 95%CI [.07, .44]), but not when it was high (B = .07, *p* = .60, 95%CI [−.19, .32]) (Fig. 3a).

For *intention likeliness*, stratified analyses revealed also that IED had a positive effect on the intention to reduce RMC when the ability to accept without judgment was low (B = .31, *p* = .002, 95%CI [.17, .45]) and moderate (B = .16, p = .002, 95%CI [.07, .26]), but not when it was high (B = .02, *p* = .79, 95%CI [−.12, .16]) (Fig. 3b).

The same pattern was revealed for *intention strength*. IED had a positive effect on the intention to reduce RMC when acceptance without judgment was low (B = .80, p = .002, 95%CI [.48, 1.12]) and moderate (B = .43, p = .002, 95%CI [.20, .66]), but not when it was high (B = .05, *p* = .76, 95%CI [−.27, .37]) (Fig. 3c). In all three regressions, multicollinearity between the mindfulness subskills was very low (*VIFs* < 1.55).

#### Intention after three months

For intention items after three months, no significant interactions between IED and the mindfulness subskills were found. All regression coefficients are depicted in Table 4. Multicollinearity between the mindfulness subskills was very low in all three regressions (*VIFs* < 1.65) and can, therefore, be ruled out as a possible reason for nonsignificant findings.

## Discussion

We did not find any mindfulness subskill to be associated with the level of congruence between implicit and explicit attitudes, which is contrary to our expectation. This finding is, however, partly in line with the findings of Hyde et al. [22] and Hofmann et al. [75], which also did not show an association between private self-consciousness and the congruence between implicit and explicit attitudes towards physical activity or different ethnicities, respectively. Both studies used a measurement comparable to the mindfulness subskill observing. Therefore, we anticipated that not only the ability to observe inner processes but also other processes, such as the ability to accept one’s inner processes as they are or non-reactance to it, are required to translate them into explicit statements. This would then ultimately lead to more congruent attitudes. However, this was not the case in the study at hand. It is possible that mindfulness is completely unrelated to the level of congruence between implicit and explicit attitudes. Yet, studies on self-esteem [10, 12] or the need for achievement [76] demonstrated that mindfulness or private body consciousness decreased dissonance between the implicitly and explicitly measured constructs. It is possible that this effect only applies to highly self-relevant and emotionally charged constructs and not to rather cognitively based constructs, such as attitudes. In order to draw more generalizable conclusions, it would be worthwhile to investigate (a) whether the present findings do or do not apply to IED in other less self-relevant domains and (b) whether mindfulness or its subskills have an effect on the congruence between implicit attitudes and only affective explicit attitudes, which are more emotionally charged.

We did not find any mindfulness subskill to moderate the relationship between IED and RMC. These null-findings could be due to different reasons. Firstly, RMC was assessed by means of self-reports. It is possible that reporting errors lead to bias in terms of the amount of consumption, thereby also leading to a distorted illustration of the relationship between IED, RMC, and the moderating role of the mindfulness subskills. A second reason could be that the mindfulness subskills simply do not moderate the relationship between IED and RMC (i.e., lack of a true effect). Whether this is the case should however be investigated by future research making use of a more objective measure of consumption. Furthermore, it would be worthwhile investigating whether these findings also apply to other types of behavior. Thereby it could be investigated whether other (health) behaviors are also positively or negatively affected by IED and whether mindfulness could function as an “antidote” in case the effect is negative.

An additional finding of the present study was that IED was related to behavior at all three measurement points, which was also the case in former studies [29, 30, 32–34, 69]. In the current study, the relationship was negative. Hence, individuals with dissonant implicit and explicit attitudes regarding RMC consume less red meat. This can be explained by Festinger’s cognitive dissonance theory [77], which postulates that individuals strive for congruence between their attitudes and behaviors. When inconsistency occurs, individuals are motivated to resolve it, as it elicits an aversive state of arousal. To do so, there are different ways such as justifying one’s behavior or cognition (e.g. “I am allowed to eat red meat once in a while”), by adding new cognitions (e.g. “Other people also eat red meat”), ignoring or denying information that conflict with existing beliefs (e.g. “There is no problem with eating red meat”) or by changing one’s behavior (e.g. “I will not eat red meat anymore”). From the present results it could be concluded that individuals mainly used the last strategy, namely behavior change, which is in line with the assumption of Festinger [77] that behavior change is the most preferred one. Furthermore, these findings are in line with the outcomes of a former review which concluded that dissonance results in behavior change [78].

Our results regarding the relationship between IED and intention suggest that there exists an additional way to deal with dissonance, which has, to our knowledge, not been identified before in the literature. The mindfulness subskill acceptance without judgment moderated the relationship between IED and intention. For people with a low and only moderate ability to accept their thoughts and feelings without judging them (including possible negative feelings resulting from IED), higher IED increased the intention to reduce RMC. For people with a strong ability to accept their inner processes as they are, IED was not associated with intention, hence no reaction was linked to IED. This finding fits nicely with the assumption that mindful people are better able to control their reactions as they have a greater acceptance of their own ‘errors’ and associated conflicts [79] as well as with the idea that by feeling their affective signals more keenly, more mindful individuals are in a better position to react to them, including an adequate control of their behavior or reaction [80]. Findings of a study of Haddock et al. [81] confirmed these ideas as more mindful people reported to feel more comfortable with holding ambivalent or dissonant views. This effect was shown for dissonance between explicit beliefs as well as between one’s implicit and explicit sexual orientation. Hence, it can be concluded that although mindfulness is not associated with more congruent attitudes, it seems to offer a skill to deal with dissonant attitudes, namely the ‘simple’ acceptance of it. This effect was not present anymore after three months and could be due to the smaller sample size at that time point.

In the present study, the non-acceptance of dissonance had a positive and thereby desirable effect on the intention to reduce RMC. Future studies should investigate in which contexts dissonance results in healthy behaviors, as it was the case in the present study, and in which contexts dissonance results in rather unhealthy behaviors, as demonstrated in former studies [32–34]. Shedding light on these questions would allow interventions to consciously induce dissonance in order to achieve certain behavior changes. However, in the context where dissonance has been shown to be maladaptive, e.g. self-esteem [15, 34], future research needs to investigate whether acceptance can be used as an alternative strategy to deal with dissonance. Attempts to alter attitudes would thereby become superfluous. Therapy approaches, such as Acceptance and Commitment Therapy [82], already entail this approach. Regarding attitude dissonance resolution, an interesting avenue for future research would be to compare the effectiveness of attitude retraining with acceptance-based approaches. Another possible avenue for future studies could be the investigation of self-compassion in the context of discrepancies. Self-compassion is an even broader construct than mindfulness as it includes the components of mindfulness, self-kindness, and common humanity. It is defined as a kind and understanding outlook toward one’s personal disappointments and struggles [83, 84]. A study on body appreciation concluded that self-compassion might work as a buffer against harshly judging discrepancies regarding body-related comparisons. Whether self-compassion might also prevent a judgmental view against attitudinal discrepancies could be investigated in the future.

Several possible limitations of this study should be taken into account. First, RMC was measured by self-report. Although based on the Food Frequency Questionnaire, a validated tool also for the assessment of meat intake [85], self-reports have been defined as less reliable as they are more prone to reporting errors than objective measurements. It is possible that the null-findings regarding our second hypothesis were a result of the usage of a self-report, which did not depict the ‘true’ relationship between IED and RMC. To conclude whether the relationship between IED and RMC is not moderated by levels of mindfulness, we encourage studies to include a more objective measurement of meat-eating behavior. A second possible limitation could be that the SC-IAT measured implicit attitudes towards red meat whereas the questionnaire measured explicit attitudes towards the consumption of red meat. It has been argued that the relations between explicit and implicit attitudes change when the category examples change [86]. The current approach was based on former studies that also assessed implicit attitudes towards an object (e.g. cigarettes) and explicit attitudes towards the behavior that entails the object (e.g. smoking a cigarette) [87, 88]. Correlational analyses showed that the two types of attitudes were, although weakly, related in our study. Whether the relationship would have been even stronger with different target stimuli used in the SC-IAT, is currently unclear and should be investigated further. One possible way to minimize the incongruity between the explicit attitude and the implicit attitude could be to assess both attitudes towards the object, hence red meat. However, a pitfall would be that explicit attitudes are not congruent with the other explicit constructs social norms, social modeling, and self-efficacy anymore, which are always assessed towards a behavior. Another way could be the usage of stimuli in the implicit task that depict the consumption of red meat more clearly (e.g. a fork with a piece of red meat pointed towards the mouth). However, it might be questionable whether the subjects are able to recognize these stimuli clearly as the consumption of red meat as other factors might be disturbing (e.g. the fork, the mouth, the face(s) of people, etc.). Future research should test the best way to minimize the incongruity between the measured attitudes.

Third, the study was conducted online, thus participants were at home in an uncontrolled environment. This appears to be an environment more prone for distractions, which, one might expect, could especially affect the SC-IAT and its outcomes. However, Houben and Wiers (2008) investigated whether an Internet-delivered IAT would yield different results compared to an IAT performed in a lab and detected no significant differences. Importantly, they even found that the IAT performed at home was more strongly related to other explicitly assessed measures as well as to the target behavior (drinking behavior) than the lab-based IAT. This supported our choice to apply this method to our sample and setting as well. Additionally, we asked participants directly after having performed the SC-IAT whether they were distracted during the task. If this was the case, their data were excluded from the analyses.

Fourth, the SC-IAT showed low test-retest stability, which is a general issue faced by researchers using implicit measures. This might be due to sensitive systematic error (e.g., learning effects or situational cues [89, 90]) or might simply demonstrate a low validity of these measures. At this point in time, it is unclear if any of these two occurrences happened and were also responsible for the null-findings. These questions are an avenue for future studies. For example, after having performed the implicit measure multiple times, participants could get asked whether they noticed a learning effect, in order to control for possible learning effects.

Fifth, acceptance of dissonance was not measured but rather inferred. Whether people who scored higher on acceptance were indeed able to better accept dissonance is unclear. Specific questions about the ability to accept dissonance could be added in future research to draw even stronger conclusions.

Sixth, the study made use of an observational design. Although its longitudinal nature is definitely a pro, an experimental approach, in which dissonance would have been induced or the level of mindfulness would have been manipulated, would allow to draw stronger conclusions in terms of causation. We are convinced, however, that the present study offers a first step and ground for this topic leading to the next logical step - a replication in an experimental setting.

## Conclusion

Mindfulness subskills are unassociated with the level of congruence between implicit and explicit attitudes toward red meat consumption. Instead, the mindfulness subskill accepting without judgment functions as a way to deal with dissonance. Future research should use this novel finding and investigate whether mindfulness can be used as a buffer in contexts where dissonance results in maladaptive behaviors. The exact relationship between attitude, attitude discrepancy, behavior, and intention appears to be complex and dependent on other variables, such as the type of behavior. To draw more generalizable conclusions, more research is needed to identify these behaviors and factors.

## Data Availability

Data are available on Open Science Framework at https://osf.io/bkp5r/?view_only=6c2e208b9e8f4354ac339c2596b85c2f.

## Abbreviations

ANEW: Affective Norms for English Words
IAT: Implicit Association Test
IED: Implicit-explicit discrepancy
RMC: Red meat consumption
RQ: Research question
SC-IAT: Single-Category Implicit Association Test
T0: Baseline measure
T1: Follow-up after one month
T2: Follow-up after three months
